# Numerical Simulations of Laser and Hybrid S700MC T-Joint Welding

**DOI:** 10.3390/ma12030516

**Published:** 2019-02-08

**Authors:** Tomasz Kik, Jacek Górka

**Affiliations:** Department of Welding Engineering, Silesian University of Technology, Konarskiego 18A, 44-100 Gliwice, Poland; jacek.gorka@polsl.pl

**Keywords:** welding, laser, hybrid, S700MC, finite element modeling (FEM), T-joint, distortions, stresses, simulation

## Abstract

This article presents examples of numerical simulations done based on the real experiments of S700MC steel T-joint laser and hybrid welding. Presented results of numerical analyses carried out using SYSWELD show the possibilities offered to contemporary engineers by modern software used to make numerical analyses of production processes. After calibration of a heat source models on the chosen examples of S700MC steel 10-mm-thick T-joint laser and hybrid welding, distributions of temperature fields, thermal cycles, distributions of individual metallurgical phases and hardness, and strains and plastic deformations in simulated processes were calculated for one selected joint from both mentioned methods. The results of the analysis allow determining both the differences in the stress distributions and their minimal and maximal values. This article also presents the benefits resulting from the use of such analyses, due to the significant savings in time and resources to be spent on the development of correct technologies for joining modern construction materials such as thermomechanically treated steels, especially given that some of the results are unavailable or very difficult to collect using conventional measurement methods.

## 1. Introduction

Welding numerical simulations are widely used for the development of new welding technology, as well as for its optimization and validation. The prediction of welding residual stress and deformation has a long history. In 1938, the first work to predict residual stresses and deformation was done by Rodger and Fletcher using analytic equations [1]. However, the inability to use the computing power of computers that appeared only after some time meant that the development of this field of knowledge had to wait. In 1970, the first welding numerical simulations using the finite element method were done by Brust, Rybicky, Barber, and Masubuchi [2,3,4]. The first industrial application of welding numerical simulations was started in 1990, mainly in the United States of America (USA), Japan, France, and Germany. During the last 20 years, the huge progress in using welding numerical solutions as industrial support was done mainly due to the big progress of computers and new solution methods, which also enable calculations of big welded constructions. The combination of new computational models with the ever-increasing computing power of computers allowed the rapid development of new applications for numerically solving issues related to the conduct of welding production processes [5].

Modern programs for numerical analysis of manufacturing processes using welding techniques, currently available on the market, create completely new opportunities for use by welding engineers. Their recent development now allows for a significant reduction in spending in this area of production preparation, both at the early design stage and technology development. However, these possibilities caused numerical analyses of welding processes to be one of the most complex calculations to carry out using the finite element method. A moving welding heat source delivers heat to the welded materials in a very specific way. Due to the specific way of material heating, the formation and development of welding strain and stress is related to a number of very closely related factors. These include conditions of fixing elements for welding, thermal and mechanical properties of materials used, type of welding technology used and set parameters, ambient temperature and pre-heating, the method of heat transfer to the environment, and many others. After the welding process, residual stresses create a balanced system of inner forces, which exist even under no external loading. The results of their impact are weld joint deformations, depending on the clamping conditions during and after the welding procedure. Therefore, the main goal for contemporary welded design is to find the optimal method of fastening welded elements during welding, as well as their cooling down. Correctly performed works allow finding a kind of compromise between the level of strain and stress in the designed joints [6,7,8,9,10]. 

**Figure 1:**
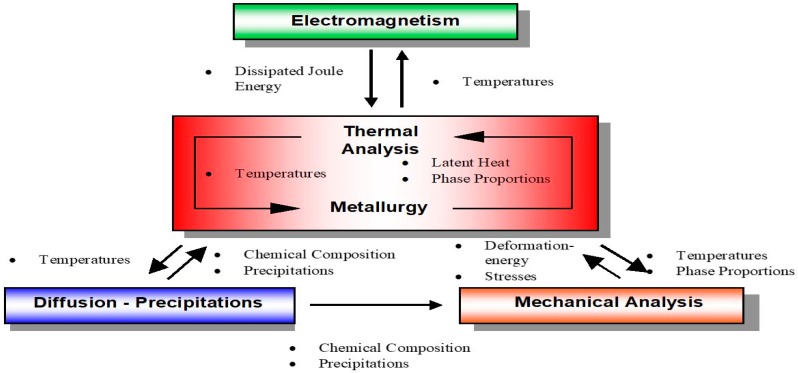
Interrelated physical phenomena [6].

Determination of the level and distribution of stresses arising after welding is a very complicated issue. The high complexity of the problem is influenced by mutual relationships among temperature distributions, the thermal expansion coefficient, heat shrinkage, and material properties, changing in time and space as a result of the influence of the welding thermal cycle. Because of the necessity for proper numerical analyses creating the possibility of taking into account the relationships mentioned above, modern computational techniques divide the analysis of welding processes into two parts. The first is a thermo-metallurgical analysis and the second is a mechanical analysis which is based on results from the previous. This division is due to the fact that changes in mechanical properties do not cause significant changes in temperature distributions. On the other hand, changes in the metallurgical phases, caused by the thermal cycle, have a significant impact on the size and distribution of stresses and strains generated during and after the welding process (Figure 1). Taking into account the greatest number of interrelations of material properties and the influence of thermal process conditions during welding allows obtaining high agreement between numerical simulations and the results of tests conducted in real conditions [7,8,9]. 

Depending on the complexity of the problem being solved, modern software for the numerical analysis of welding processes has the ability to perform calculations using a variety of methods. The most commonly used are as follows [5,6,7,8,11,12,13,14]: continuous analysis of the welding process (transient method), “local–global” method, and shrinkage analysis.

In each of these methods, the user gains access to computational capabilities adapted to the problem being solved and the expected results. The continuous analysis of the welding process (transient analysis), also called the “step by step” method, is based on calculations related to the moving mathematical model of the heat source. This analysis is divided into two parts: calculations of thermal phenomena and related metallurgical changes, and mechanical phenomena (i.e., distributions of stresses and strains, etc.). Taking into account the performance of calculations at each subsequent time point, the user receives a powerful set of thermometallurgical and mechanical data concerning the simulated process after completing the analysis. The price of such a solution is the extended duration of calculations. This is the main reason why the calculation of large weldments with many welds uses other computational techniques [1,2,3,4,5,6,7,8,13]. One of them is the modification of the “transient” technique called the “macro bead deposit” method (MBD). In this method, we use a properly prepared thermal cycle immediately on one or several areas (elements) of the model at the same time. The actual welding trajectory is divided into sub-areas, such that the order and the direction of welding are maintained. The number of these sub-areas and the time step are defined based on the technological parameters of the welding process, as well as the experience of the person working with this method. Although this technique requires much more experience than the person preparing the model for calculations, its application significantly shortens the calculation time. This allows performing numerical analyses of large, complex structures while maintaining high consistency of the results of these analyses with reality [5,6].

The “local–global” method is a particularly important technique in the case of numerical analyses of very large structures with a large number of welded joints. This method is used in situations where the standard (transient) method or even MBD is not possible to carry out. The main idea of the “local–global” technique is the assumption that the welding process leads to local changes in the distribution of stresses and plastic deformations, whereas the effect of this on a global scale is the defined state of deformation. The local effects of the welding process are determined in this case by means of calculation models of individual welded joints using the previously mentioned techniques. The results of local analyses are then transferred to the global model (whole structure) in order to determine the total deformation of the structure. A certain limitation of this calculation method is that the resultd of the analysis are only the deformations of the structure and internal forces and moments in the specified mounting conditions. Stress levels and distributions of individual metallurgical phases are determined only in local models [5,6,12,13].

The final mentioned method, known as the “shrinkage method” allows performing quick structural deformation analysis with a very large number of welded and welded joints. In addition, it is possible to include, for example, the history of sheet metal pressing processes, the positioning method and fastening of the connecting elements, the springing effect, the fixing bases, various joining techniques, and others. As mentioned earlier, this technique is based on the numerical analysis of contraction. It is a tool that allows determining the deformation of a complex structure caused by the welding process in a very short time, and very quickly and efficiently creating a welding plan, determining the method and sequence of fastening elements, and optimizing the welding sequence. An additional advantage of this calculation method is the fact that it is possible to use “shell” elements to build the so-called shell model (significant acceleration of calculations). There is also the possibility of calculations on models built of three-dimensional (3D) solid types of elements and models combining “solid” elements with elements of the “shell” type [5,6].

The present problem of numerical welding simulations is that calculations of temperature fields, metallurgical phases, thermal cycles, as well as the the strains, stresses, and distortions connected with them, are strongly connected with many factors which describe the process and use a proper methodology. Advantages of using new possibilities in simulation techniques are also strictly connected with the continuous development of new construction materials, which usually have significantly higher requirements also for the techniques of joining them. Moreover, it is not only directly about the quality of joints, but also the production capabilities, manufacturing costs, and the possibility of precise control of the technological process [6,11,12,13]. 

A very good example of this is the current use of very popular laser heat sources. Of course, they deliver many advantages such as a low heat effect, causing a narrow heat-affected zone (HAZ) and limited deformations connected with high process efficiency. However, it is also known that a very precise preparation is needed of the welded element edge, and, with an increase in the dimensions of welded elements, there are also problems with positioning [10,11,12]. Additionally, if we attach a very short thermal cycle to these problems, then, in many modern steels, this may be related to the existence of zones in which the structures obtained as a result of welding will be fragile and will not provide adequate plasticity of joints. Another disadvantage of these modern heat sources is a relatively small efficiency. It is especially important if we also take into account the current energy problems of the world in which we live, as well as striving to reduce costs at every stage of the production process, wherever possible [15,16,17,18,19,20,21,22,23,24]. 

The comparison of the mentioned advantages and disadvantages of the classical (arc) methods of welding forces us to look for new solutions that combine the advantages of using high-density heat sources with methods that allow maintaining high strength and operational properties of modern and, therefore, more expensive construction materials. A certain solution to this problem, combining the advantages of laser welding while increasing the efficiency of the process is called hybrid welding (HLAW—hybrid laser arc welding) [24,25,26,27,28]. It consists of the use of two independent heat sources, such as a laser beam (most commonly a solid-state YAG laser) and an electric arc of the GMAW (Gas Metal Arc Welding) method. The addition of the arc welding process ensures the correct filling of the groove, decreasing the requirements for precise preparation of the edges of welded elements. It can be said that this method combines the laser welding speed with the advantages of arc welding, allowing a reduction in the number of beads compared to traditional methods [28,29,30,31,32]. 

However, the use of two significantly different heat sources brings with it the need to control a much larger number of parameters at the same time. The use of numerical techniques at the design and preparation stage of welding technology is particularly recommended in this case. This way of thinking by a modern engineer leads to a significant increase in the quality of manufactured products [33,34].

## 2. Methodology and Assumptions

### 2.1. Laser and Hybrid T-Joint Welding Experiments

This study aimed to determine the influence of the thermal cycle of the laser and hybrid welding process on the structure and stress distribution in T-joint welds of S700MC steel plates with a thickness of 10 mm. The article presents selected results of numerical analyses of the laser and hybrid welding process of the same type of joint. As a result, it was possible to show differences in the metallurgical phases, hardness, and stress distributions in both cases. S700MC, used as a base material, is a hot-rolled, high-strength, low-alloy steel and combines high strength with outstanding formability and consistent quality (Table 1). As provided by the producers and supplier, it also delivers exceptional weldability for fast and efficient processing. However, along with the change in the thickness of the welded elements, as well as the use of welding methods with a very sharp thermal cycle, unexpected problems are associated with the weldability of components made of this type of steel, as well as with the expected functional properties of the welded joints [21,24,29,30,35].

The results of welding tests were used not only for the selection of best welding parameters, but also to provide input data for the calibration of computational models calculated in the VisualWELD environment (SYSWELD release 14 produced by ESI Group, Paris, France). Welding parameters determined on the basis of tests are shown in Table 2 and Table 3. 

In the hybrid welding (laser beam—GMAW) process of T-joints made of 10-mm-thick steel S700MC, solid wire GMn4Ni1.5CrMo with a diameter of 1.2 mm was used. The chemical composition and the properties of the weld deposit are presented in Table 4.

### 2.2. Model and Assumptions for the Numerical Simulations

In the presented numerical simulation of laser and hybrid T-joint welding, the VisualWeld (SYSWELD) software package was used. It is a modern, widely used commercial simulation software for welding and heat treatment processes. Temperature fields were calculated based on Fourier’s differential formula base. For calculations, it was, therefore, necessary to acquire the temperature dependence of the heat conductivity coefficient, specific heat, and density [6,11,12,36].

(1) $$\frac{\partial T}{\partial t}=\frac{\lambda }{C\times \rho }\left(\frac{\partial^{2}T}{\partial x^{2}}+\frac{\partial^{2}T}{\partial y^{2}}+\frac{\partial^{2}T}{\partial z^{2}}\right)=a\nabla^{2}T,$$

where *T* is the temperature (K), *t* is the time (s), *x*, *y*, and *z* are point coordinates (m), *a* is the thermal diffusivity coefficient (m^2^·s^−1^), *λ* is the heat conductivity coefficient (W·m^−1^·K^−1^), *C* is the specific heat (J·kg^−1^·K^−1^), and *ρ* is the mass density (kg·m^−3^).

In SYSWELD, the modified heat conduction equation is used as follows:

(2) $$\left(\underset{i}{\sum }P_{i}{\left(\rho C\right)}_{i}\right)\frac{\partial T}{\partial t}-\nabla \left(\left(\underset{i}{\sum }P_{i}\lambda_{i}\right)\nabla T\right)+\underset{i<j}{\sum }L_{ij}\left(T\right)\times A_{ij}=Q,$$

where *P* is the phase proportion, *i*,*j* are phases indexes, *Q* is the heat source, *L_ij_*(*T*) is the latent heat of *i* → *j* transformation, and *A_ij_* is the proportion of phase *i* transformed to *j* in a time unit.

A correctly performed numerical analysis of the welding process involves the appropriate definition of the method of introducing heat to the material. The available literature describes many possible mathematical descriptions of this issue. It lists, among others, the two-dimensional (2D) Gaussian surface heat source model, the Goldak double-ellipsoidal heat source model, and the three-dimensional (3D) Gaussian conical heat source model. Each of the mentioned models of heat source finds its application in the modeling of selected welding processes. The 2D-Gaussian model performs well in the modeling of surface treatment processes, while the Goldak model is a very useful tool when the process is carried out using “melt-in welding”. The 3D-Gaussian conical model reflects very well the conditions in which heat sources with high power density (laser or electron beam) are used [6,7,8,36,37]. 

**Figure 2:**
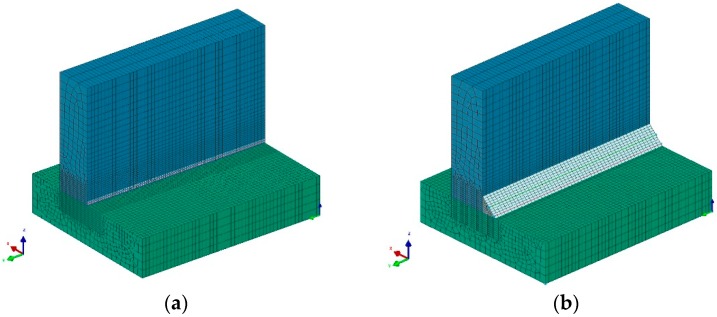
View of calculation three-dimensional (3D) solid models for laser and hybrid welding simulations: (**a**) laser and (**b**) hybrid.

The prepared calculation 3D model consisted of 45,250 3D solid elements with 48,654 nodes in the case of the laser welding model, and 47,000 3D solid elements with 50,082 nodes in the case of hybrid welding. The mesh was concentrated in the weld area to increase calculation accuracy (Figure 2). Boundary conditions related to clamping conditions during welding were set to simulate welding without any additional clamps. For boundary conditions corresponding to heat dissipation to the environment, it was assumed that, on each external surface, the model was cooled to ambient temperature (20 °C). All calculations were performed using the “transient” calculation method. This means that analyses were continuous with time steps defined automatically by the solver, based on mesh dimensions and the size of heat source models. In the case of hybrid welding, the arc heat source was placed 4 mm behind the laser beam. 

Heat source models in VisualWeld (SYSWELD) are described by a volume density of energy applied to elements *Q*(*x*,*y*,*z*). Additionally, this heat source model moves along the welding trajectory. All heat source parameters (i.e., energy, efficiency coefficient, torch shape, and others) can be included in a Fortran type function which is used for the description. The volumetric density of energy defined by this Fortran function on the current point depends on the distribution of density around the center of the source and trajectory. 

**Figure 3:**
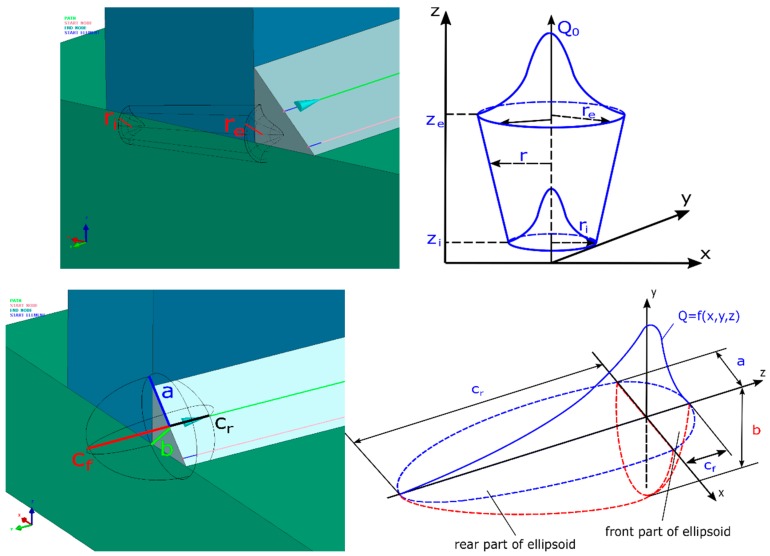
View of used heat source models—3D conical and double-ellipsoidal (Goldak’s) model.

In the present work, double-ellipsoidal (Goldak’s model) and 3D conical models were selected (Figure 3). The 3D conical model is a type of source which is used for the correct design of a welding simulation using a laser or electron beam characterized by high power density. From a parametric point of view, the model is determined by the power of the heat source, its radius, and the depth of penetration [6]. Mathematically, it can describe the conical model with Equations (3) and (4) (Figure 3).

(3) $$q\left(x,y,z\right)=q_{0}exp\left(-\frac{x^{2}+y^{2}}{r_{0}^{2}\left(z\right)}\right),$$

(4) $$r_{0}^{2}\left(z\right)=r_{e}+\frac{r_{i}-r_{e}}{z_{i}-z_{e}}\left(z-z_{e}\right),$$

where *q*_0_ is the heat flux density, *r_e_*, *r_i_* are the 3D cone radius dimension parameters, and *z_e_*, *z_i_* are the 3D cone length parameters.

Firstly, Equation (3) describes the heat transfer to the material depending on the coordinate data. It is supplemented by Equation (4), defining the change in radius along the depth. The conical model is mainly used to simulate laser welding and electron beams, but it can also be used for other heat sources, such as plasma arc during keyhole technique welding. The double-ellipsoidal model (Goldak’s model) is a heat source model which can be also described by Equations (5) and (6) (Figure 3). The efficiency of the heat transfer into the parent material is given by the applied welding method. The geometry of the double-ellipsoidal model can be modified by changing coefficients a, b, and c contained in the equations. By changing these parameters, we have the advantage of greater flexibility in the modeling of a heat source shape. It is important that VisualWeld (SYSWELD) enables the capability to introduce a power density function applied to the structure Q_R_ (W/mm^3^). Because of it, the energy is divided into *O_f_* and *Q_r_* values. The first value is the heat energy density in the front half of the ellipsoid (maximum source frontal intensity), and the second is that in the rear part (maximum source rear intensity) (Figure 3).

Transferred heat is described by the equations below [6]. 

For the front part of the heat source model, it can be described as

(5) $$Q_{f}\left(x,y,z\right)=\frac{6\sqrt{3}f_{f}Q}{abC_{f}\pi \sqrt{\pi }}exp\left(\frac{-3x^{2}}{a^{2}}\right)exp\left(\frac{-3y^{2}}{b^{2}}\right)exp\left(\frac{-3z^{2}}{c^{2}}\right),$$

And, for the rear part of heat source model, it can be described as

(6) $$Q_{r}\left(x,y,z\right)=\frac{6\sqrt{3}f_{r}Q}{abC_{r}\pi \sqrt{\pi }}exp\left(\frac{-3x^{2}}{a^{2}}\right)exp\left(\frac{-3y^{2}}{b^{2}}\right)exp\left(\frac{-3z^{2}}{c^{2}}\right),$$

where *Q_f_*, *Q_r_* is the heat introduced into the front and rear part of the model, *Q* is the total power source, *a* is the width of the molten pool, *b* is the depth of the molten pool, *c_f_*, *c_r_* is the length of the front and rear part of the molten pool, and *f_f_*, *f_r_* are constants which influence energy flow intensity into the material.

Another advantage of the VisualWeld (SYSWELD) package is the possibility of changing the thermal load area shape and achieving the possibility of the very precise modeling of fusion line geometry. 

## 3. Results of Laser and Hybrid Numerical Simulations

After model preparations, the calibration of the described heat source models was done. To achieve the best correlation with real welding tests (comparison of molten areas on macro views and registered thermal cycles), 3D numerical models were calibrated using the heat input fitting module to optimize the virtual molten metal pool shape. Final values of parameters used in the finite element modeling (FEM) analyses are presented in Table 5. Due to THE large number of results, one of the analyses for the laser and hybrid welding process was selected for comparison. Used parameters for the laser welding analysis correspond with the parameters used for joint LAS7 (Table 2). In the case of hybrid welding, parameters used in numerical simulations were set as for the HYB1 joint (Table 3).

**Figure 4:**
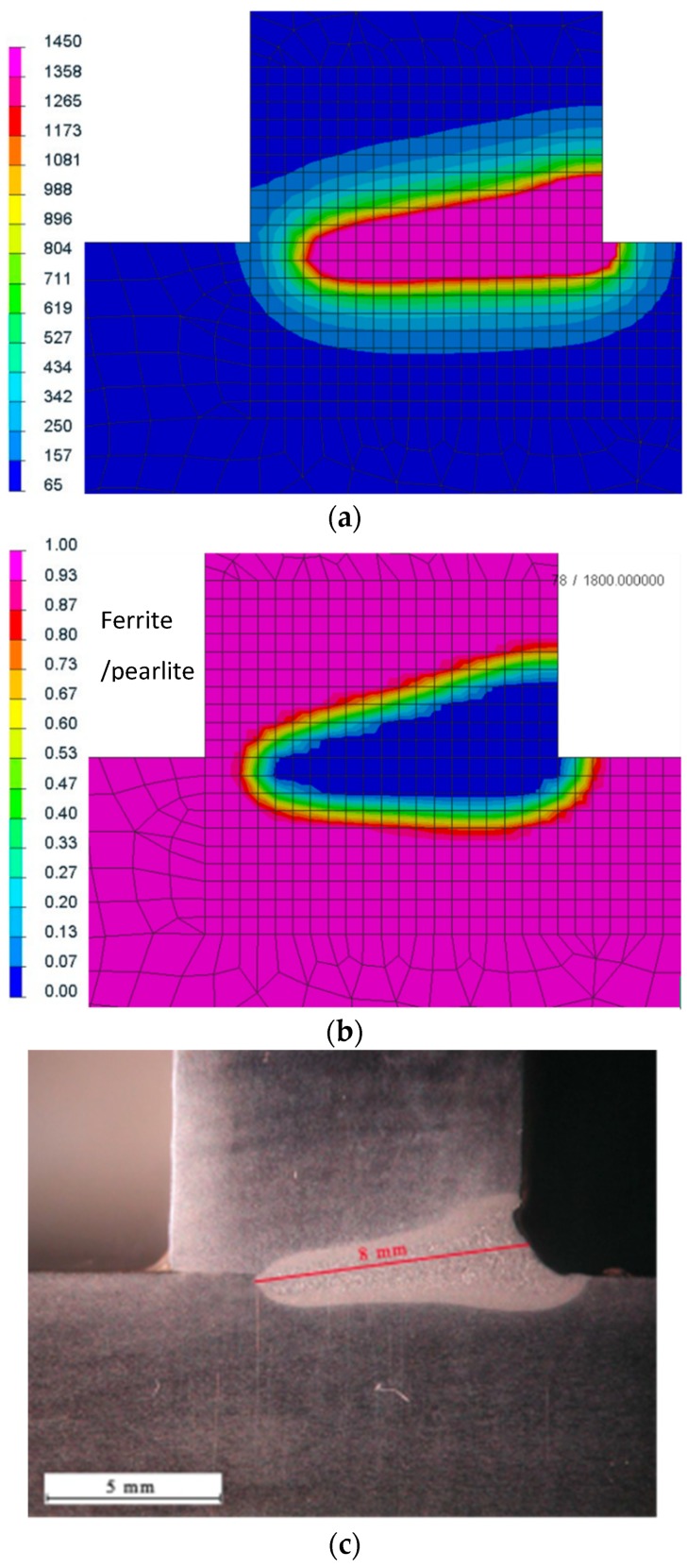
Laser welding (**a**) temperature field distribution, and comparison of (**b**) a ferrite/pearlite as an initial phase distribution with **(c)** a macro-section of a welded joint (the blue color is the estimated melted zone/weld area; cross-sections were made in the half-length of the joint).

**Figure 5:**
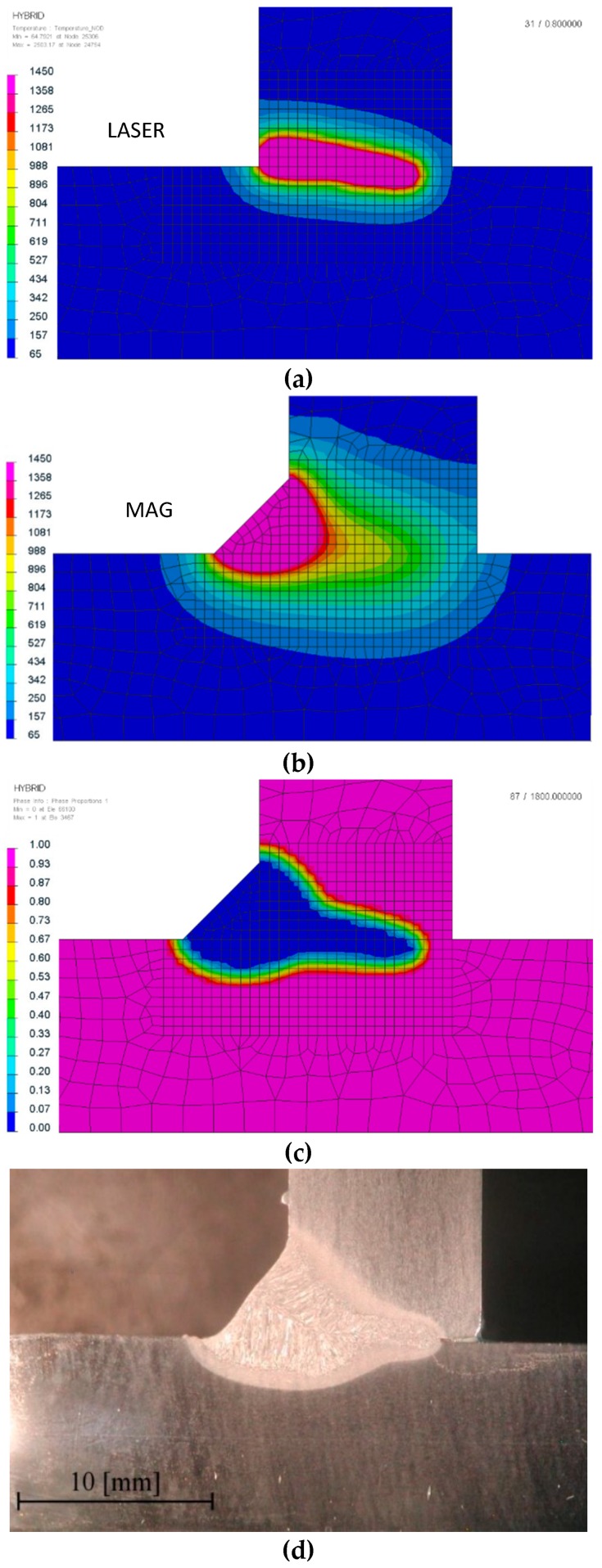
Hybrid welding temperature field distribution separately for (**a**) the laser and (**b**) the GMAW arc, and comparison of a (**c**) ferrite/pearlite as an initial phase distribution with (d) a macro-section of a welded joint (the blue color is the estimated melted zone/weld area; cross-sections were made in the half-length of the joint).

**Figure 6:**
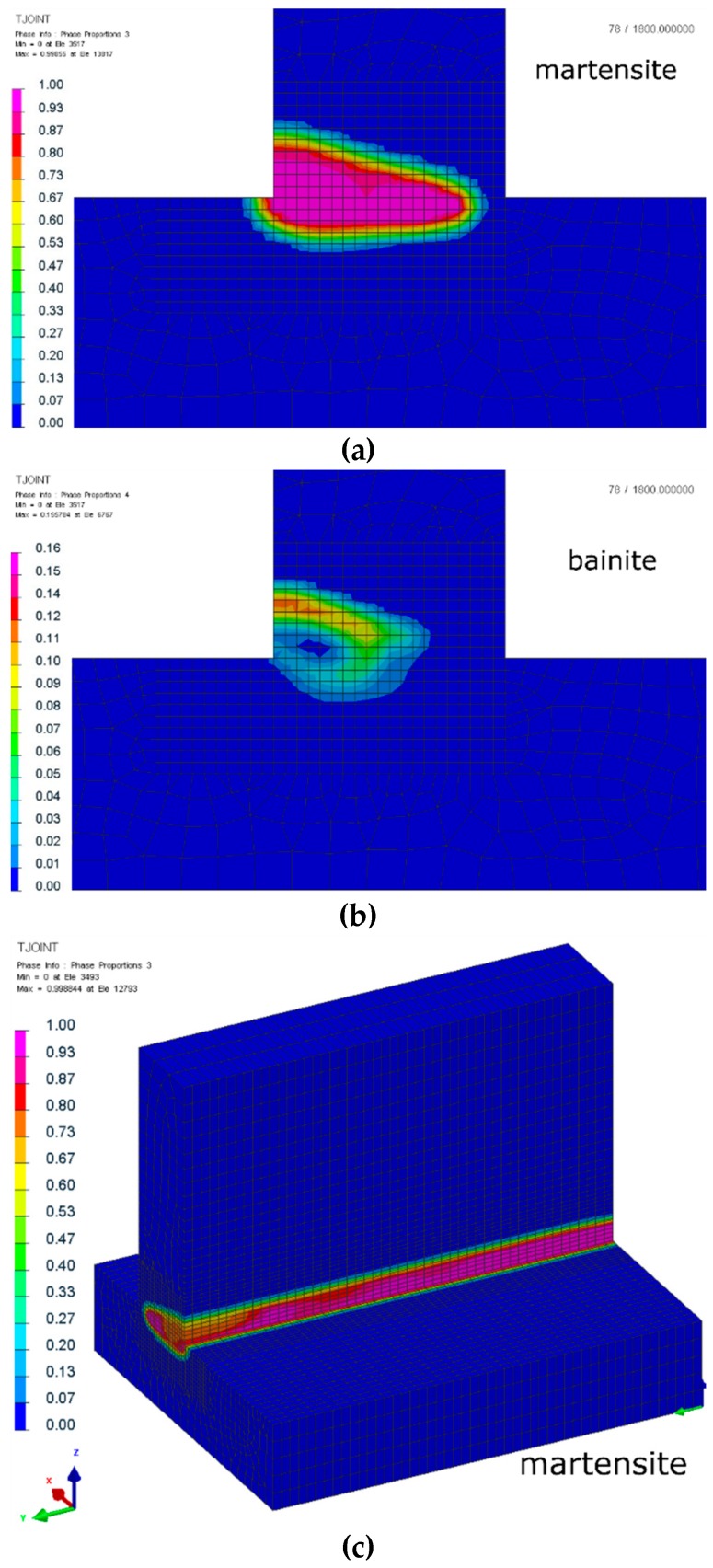

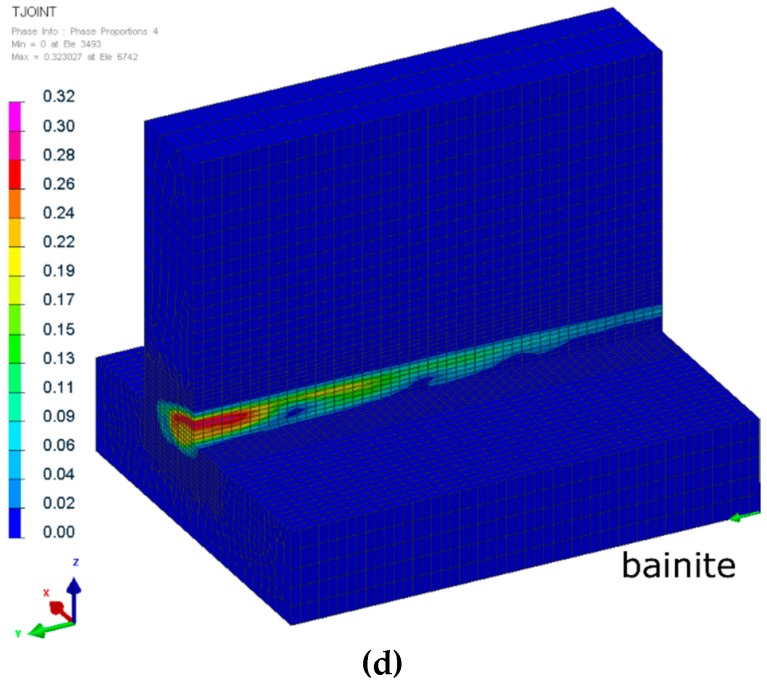
Distribution of (**a**,**c**) martensite and (**b**,**d**) bainite phases after laser welding of S700MC steel T-joints with a thickness of 10 mm (cross-sections were made in the half-length of the joint).

**Figure 7:**
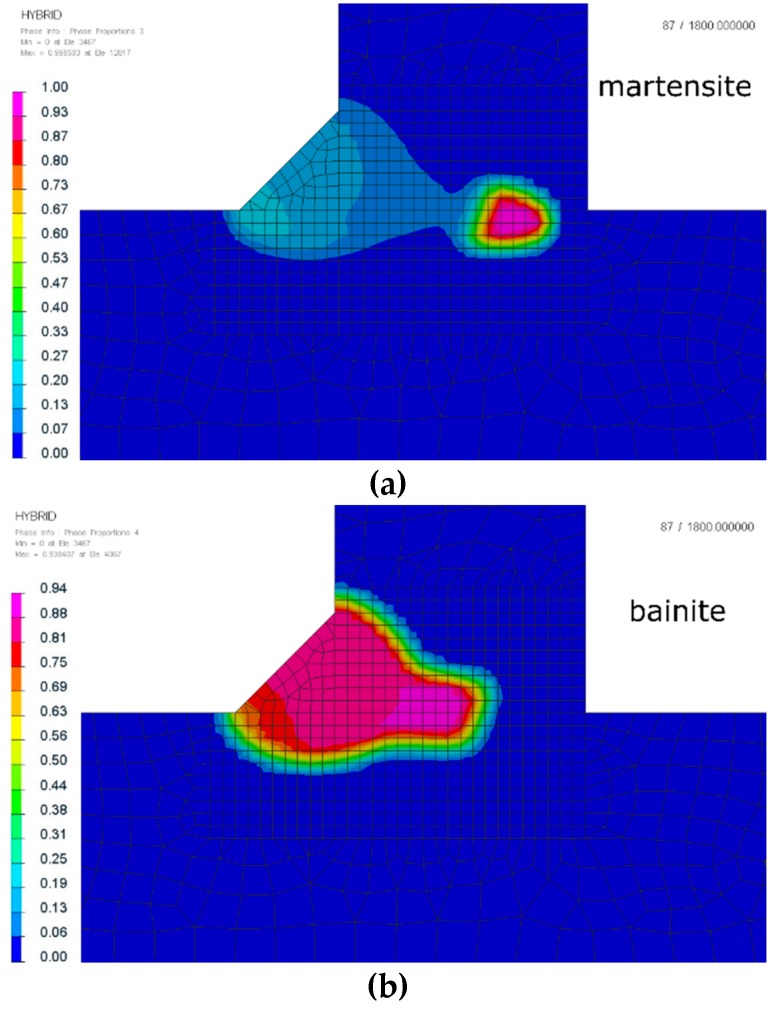

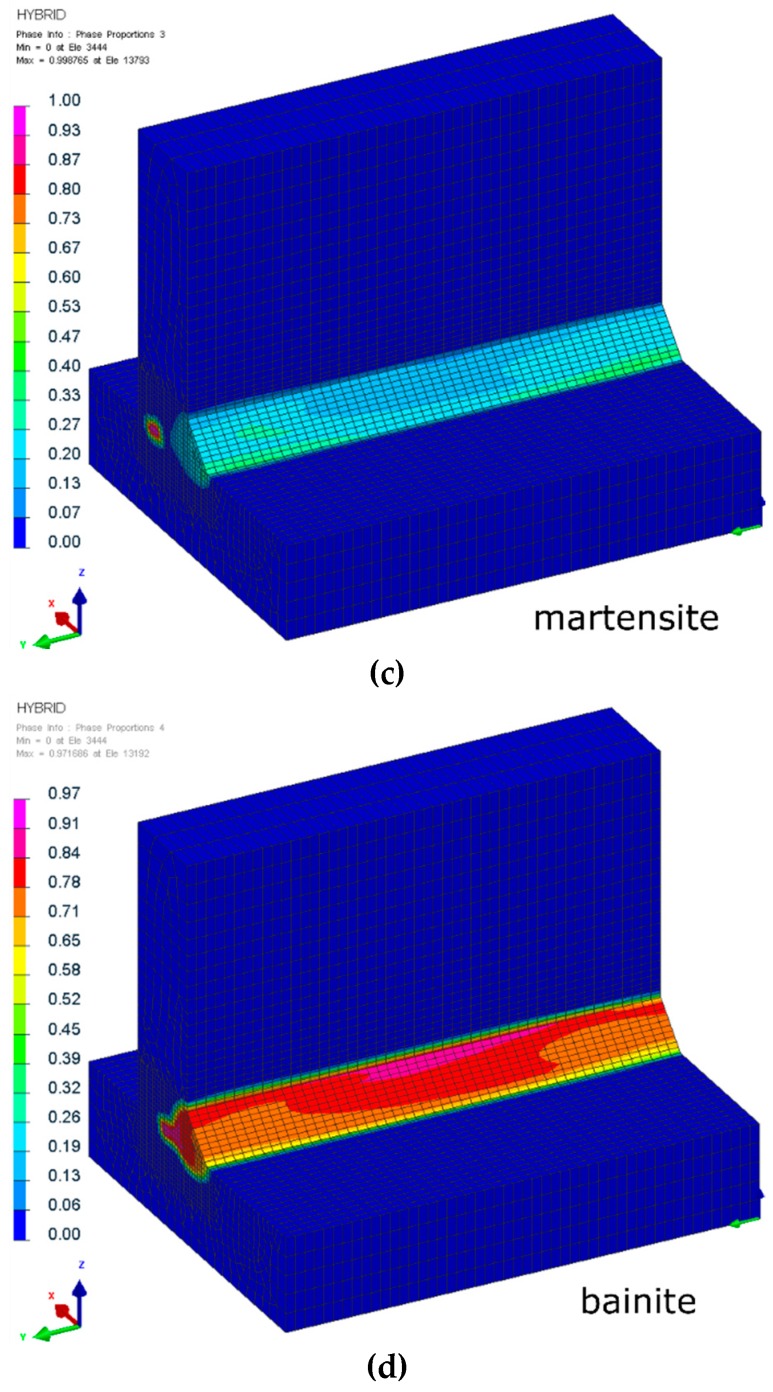
Distribution of martensite (**a**,**c**) and bainite (**b**,**d**) phases after hybrid welding of S700MC steel T-joints with a thickness of 10 mm (cross-sections were made in the half-length of the joint).

The above parameters were used in the main simulations. The thermo-metallurgical analysis allows not only determining values and temperature distribution, but also determining each metallurgical phase in calculated joints after welding and cooling to the ambient temperature (Figure 4, Figure 5, Figure 6 and Figure 7). They were calculated based on Leblond’s model which generalizes Johnson–Mehl–Avrami type kinetics. Martensitic transformation was calculated based on the Koinstinen–Marburger formula. In metallurgical phase calculations, SYSWELD uses the kinetic transformation at constant elevated temperature as a modified Leblond’s model. There was a visible difference between both (laser and hybrid) distributions of the temperature fields. Heat range (understood as the area of heat influence) in the hybrid method was bigger than that using the laser. The distribution of the initial phase, which is possible to calculate, gives the information about the material that undergoes metallurgical changes as a result of the interaction of the welding thermal cycle. Of course, it is clear that the transformed part of these figures is bigger than the real melted area, and also includes the heat-affected zone (HAZ). However, the shape of this distribution gives the main information about the weld geometry (Figure 4 and Figure 5). 

Analyses of the other calculated metallurgical phases indicate that, in the case of laser welding, the amount of martensite was almost 99% in some areas and the maximum amount of bainite was just about 32% (in the middle-length of the joint, it was about 15%) (Figure 6). The connection of a very short laser thermal cycle and the thickness of the welded elements resulted in a high cooling speed in the area of the weld and the HAZ. 

In the case of hybrid welding, the cooling rates were lower due to the presence of additional heat from the MAG heat source. This additional portion of heat coming from the MAG electric arc resulted in a significant decrease in cooling speed, and it is clearly visible in the thermal cycle graph, in addition to the distribution of bainite and martensite at the distribution of metallurgical phases. Of course, the maximal amount of the bainite was about 97% in the area only heated with the laser. However, in the most areas of the weld (remelted or heat affected by MAG arc), the maximum amount of martensite did not exceed 20–30%. The distribution of bainite also corresponded to the decrease in cooling rates mentioned above. In the area of the MAG heat source, the maximum range of bainite was about 97% (Figure 7). 

**Figure 8:**
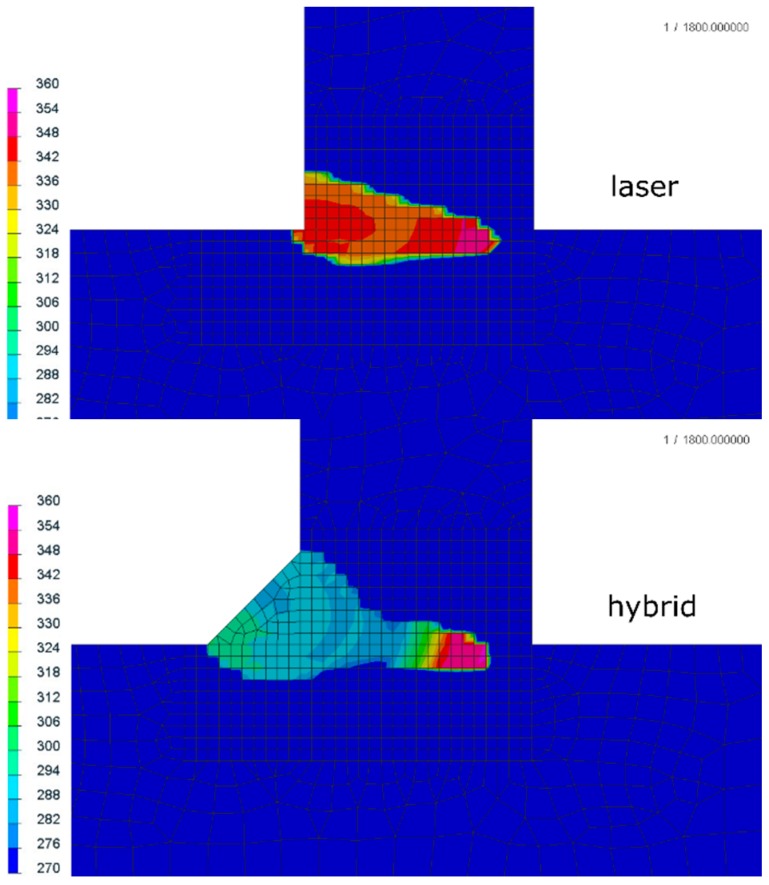
Distribution of calculated Vickers hardness after laser and hybrid welding of S700MC steel T-joints with a thickness of 10 mm (cross-sections were made in the half-length of the joint).

Using a coupled thermo-metallurgical analysis gave us the possibility of hardness distribution calculations. They were calculated based on the metallurgical phases, the chemical composition, and the cooling speed rates. It is visible that the hardness values confirmed the martensite and bainite distribution presented above (Figure 8). Calculated results were also compared with the real hardness measurements, as shown in Table 6.

**Figure 9:**
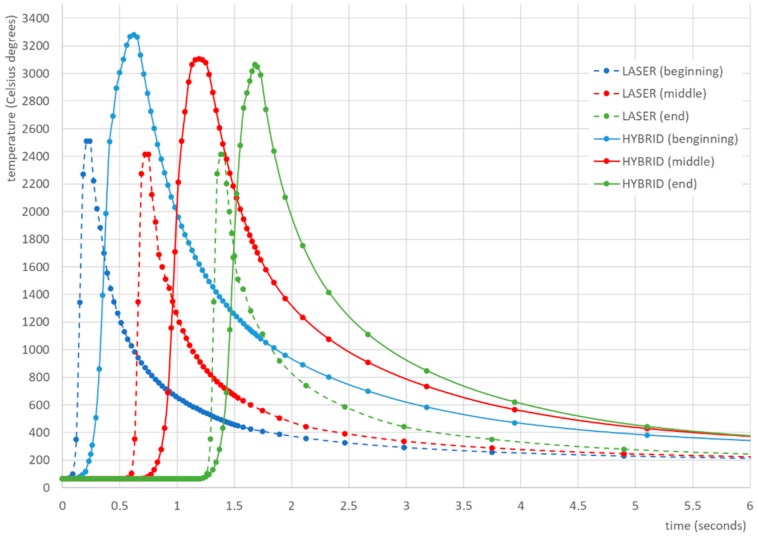
Calculated thermal cycles for laser and hybrid welded S700MC steel T-joints with a thickness of 10 mm.

The heat cycles calculated in the middle-length of the weld for both analyzed welding methods indicated that the typical t8/5 time (time for the temperature dropping from 800 to 500 °C, inversely proportional to the cooling rate) for laser welding was about 0.65–0.7 s. For comparison, the same time for hybrid welding was about 1.3–1.5 s. Thermal cycles separated to determine the t8/5 time were taken from the beginning, middle, and end of the weld length (Figure 9).

**Figure 10:**
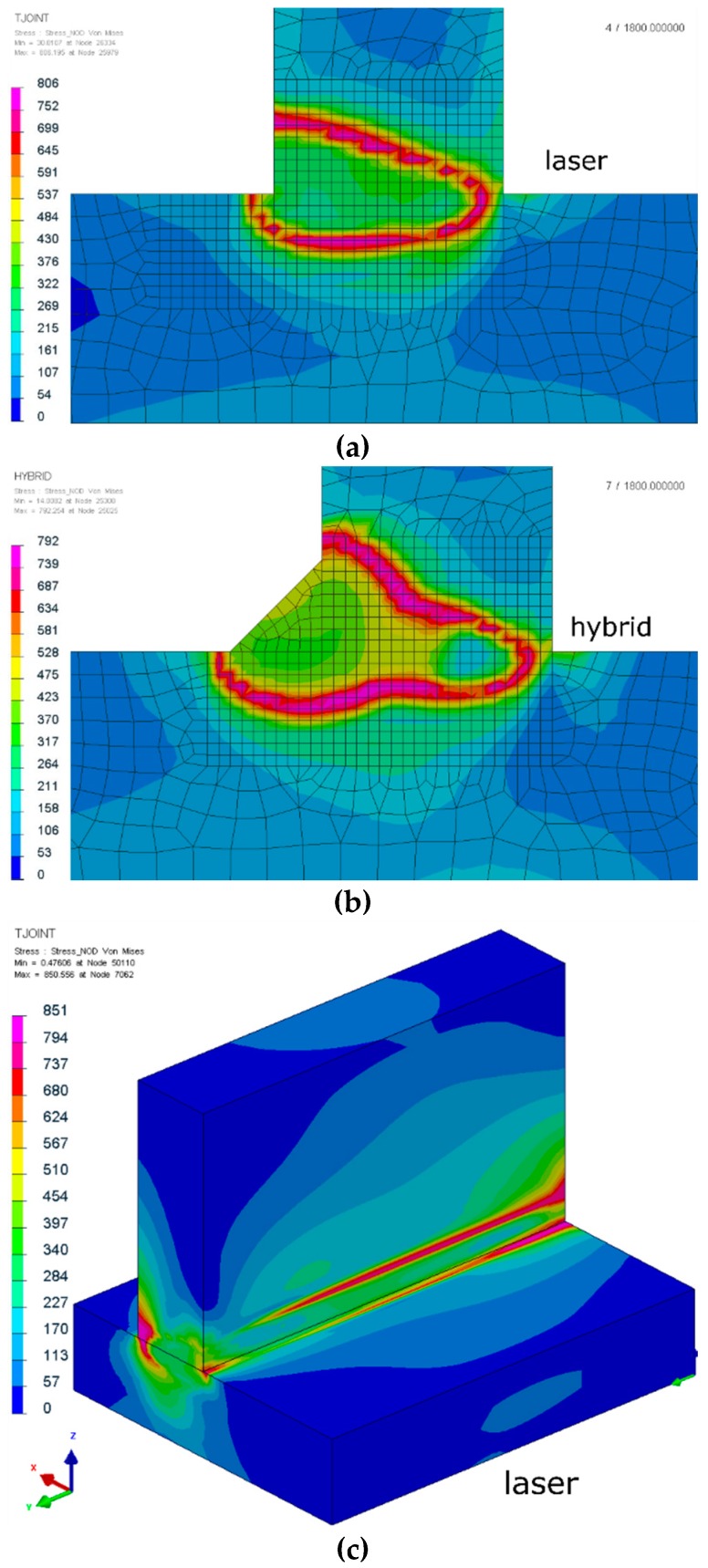

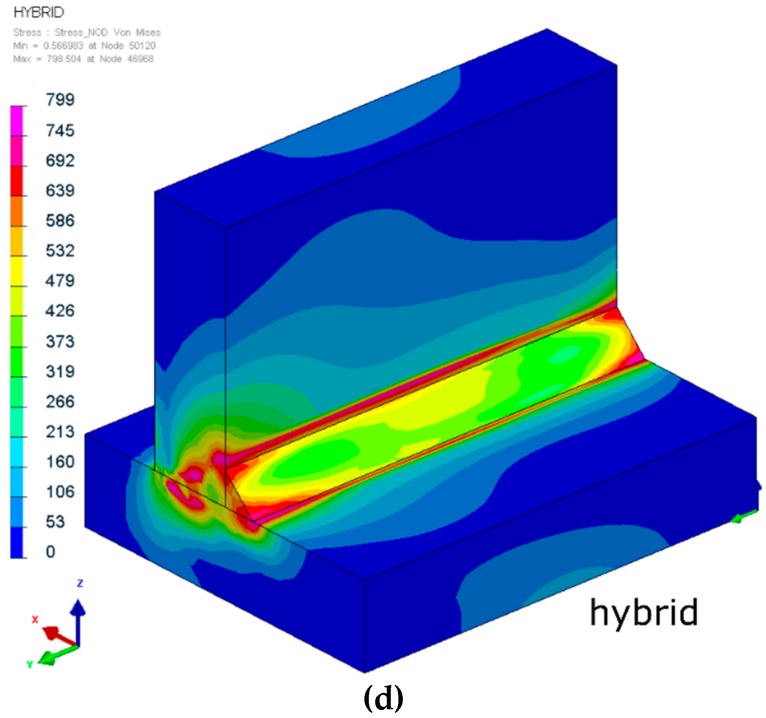
The von Mises stress distributions of laser (**a**,**c**) and hybrid (**b**,**d**) welded S700MC steel T-joints with a thickness of 10 mm (cross-sections were made in the half-length of the joint).

It is obvious that the previously discussed distribution of metallurgical phases and the influence of different thermal cycles will also be reflected in the distribution and values of calculated residual stresses in the mechanical analysis. This additional information is very often valuable for engineers and very difficult or impossible to collect during real welding tests. In the calculated examples, differences between maximum von Mises stresses were also visible. For laser welding, the maximum value was about 850 MPa, and the areas of higher stresses were located in the area of the fusion line (Figure 10). For hybrid welding, the maximum von Mises stresses were also located in the area of the fusion line, with particular emphasis on the lower part of the joint where the main interaction of the laser beam took place. The occurrence of a ferrite phase, arising as a result of the heat of the MAG method on the martensitic structures formed as a result of the thermal cycle of the laser beam, resulted in lower von Mises stresses at a maximum level of about 799 MPa (Figure 10). 

**Figure 11:**
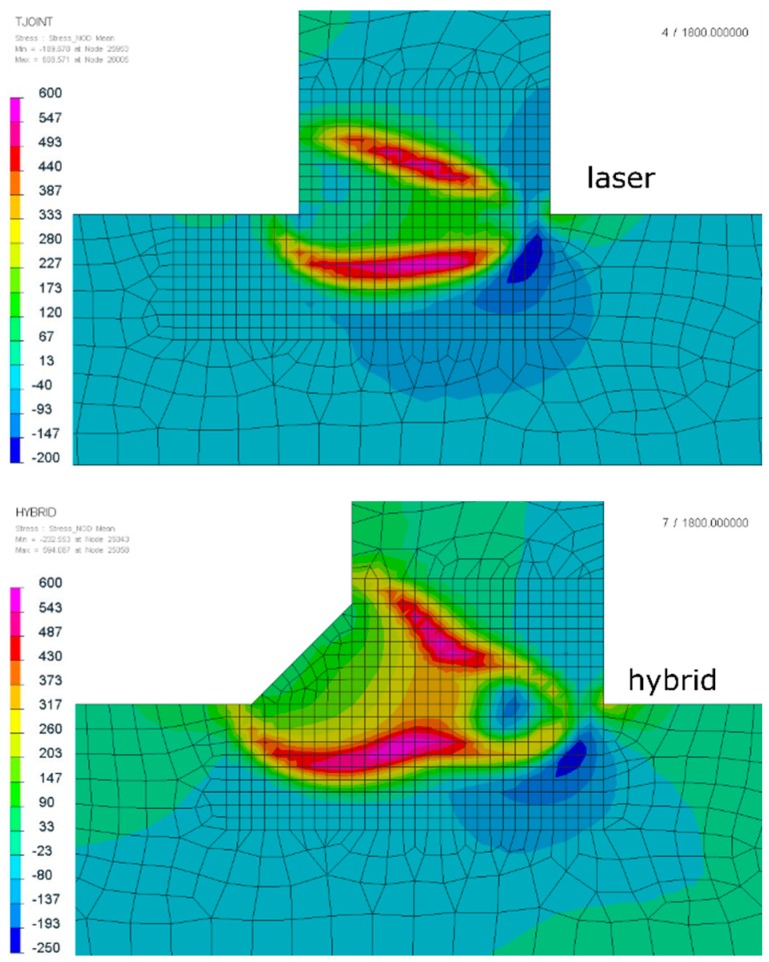
Residual stress distributions of laser and hybrid welded S700MC steel T-joints with a thickness of 10 mm (cross-sections were made in the half-length of the joint).

**Figure 12:**
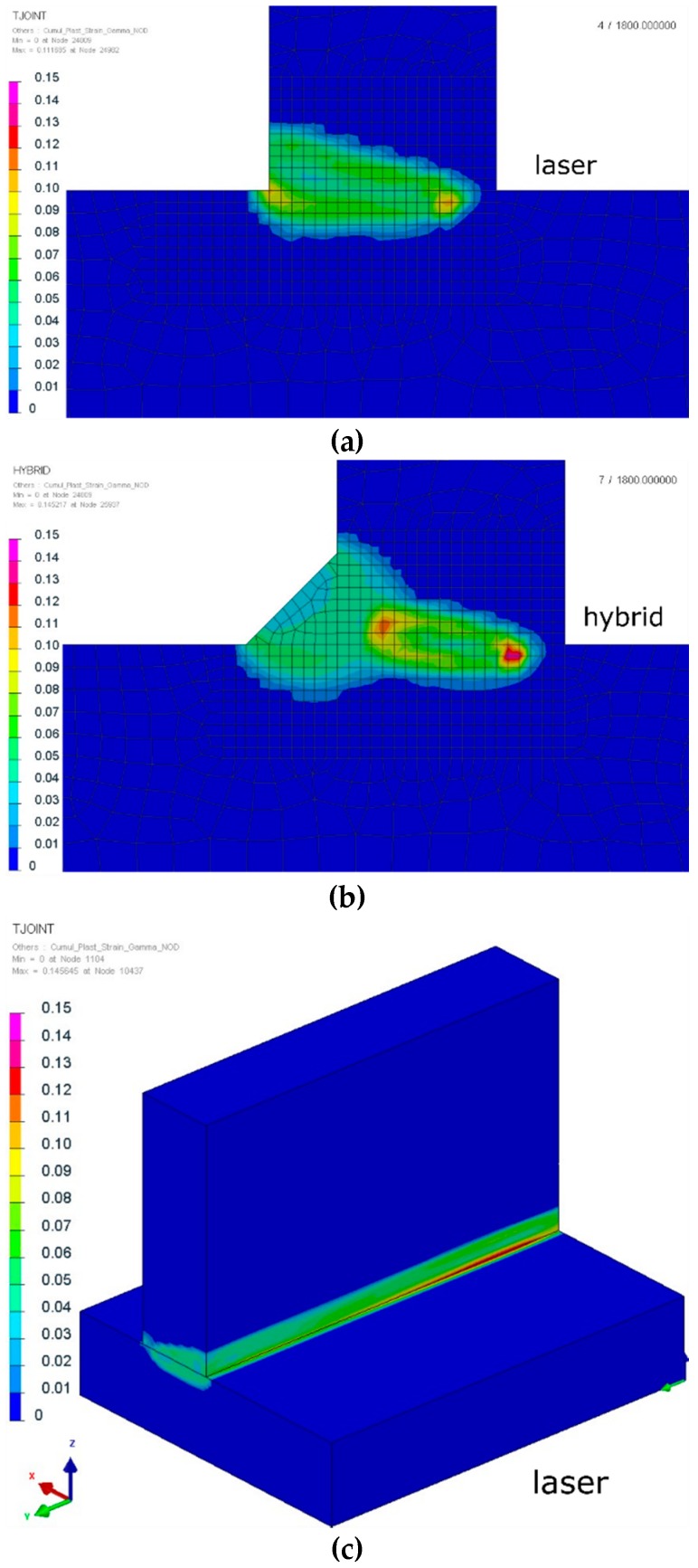

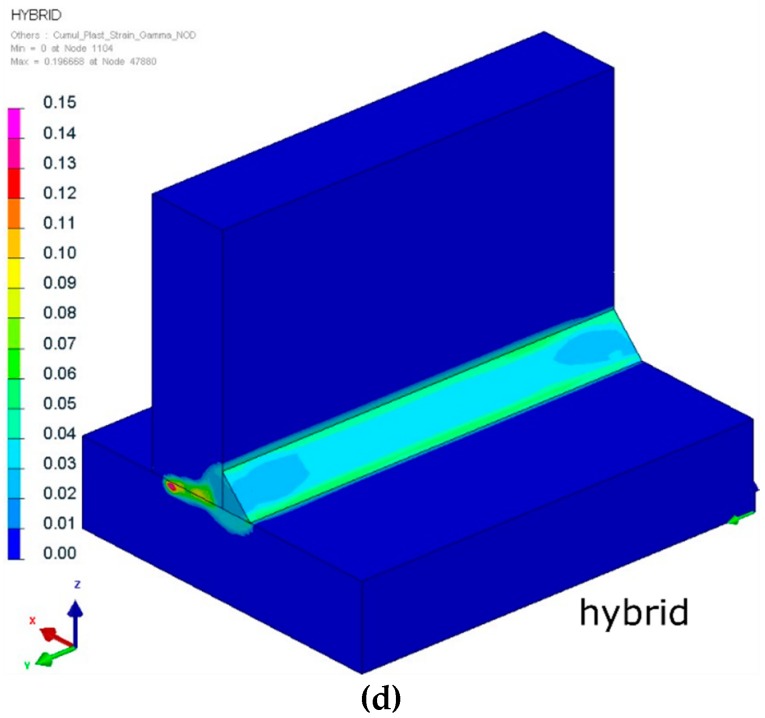
Cumulative plastic strains of the (**a**,**c**) laser and (**b**,**d**) hybrid welded S700MC steel T-joints with a thickness of 10 mm (cross-sections were made in the half-length of the joint).

Analyzing the residual stress distribution, it is clearly visible that stresses also concentrated near the fusion line (Figure 11). The values and the location of both tensile and compressive stresses were similar with some small differences. Maximal values of tensile stresses were located in the HAZ (Figure 11). In SYSWELD, it is possible to calculate cumulative plastic strains as a measure of how much yielding strain occurred. Comparing the values, they were almost the same for both methods; however, it is visible for the hybrid welding that the maximum values were located at the “bottom” of the weld (laser influence area), compared to the laser welded joints, where the areas of maximum plastic strain (about 15%) were also located at the surface of the weld (Figure 12). 

## 4. Conclusions

The presented results of conducted numerical analyses of laser and hybrid welded S700MC T-joints with a thickness of 10 mm indicate that it is possible to achieve substantial additional information about the welding process without carrying out real tests.

The presented results of numerical analyses for selected calculation examples showed that, after precise calibration of heat source models, it was possible to obtain results of thermo-metallurgical analyses coincident with the results of actual welding tests. Both in the case of the analysis of the distributions of temperature fields and the initial distribution of the metallurgical phase, their comparison with the obtained macroscopic defects showed a high degree of similarity in the presented data. This part of the analyses is very important and often requires a lot of time in order to fine-tune the data entered into the numerical analysis, so that both the cooling rates and the maximum values of temperature achieved are close to real values. However, it should be remembered that the model is always a kind of approximation or simplification of reality; hence, some minor discrepancies are possible and did occur. However, they are very easy to explain using the engineering knowledge of the welding personnel, upon understanding the specificity of the numerical simulations. 

As already mentioned above, the calculated distributions of the remaining metallurgical phases can be confirmed using the values taken from the calculated thermal cycles. In the case of laser welding, a very short thermal cycle was reflected in the high martensite content in the joint and HAZ. An almost doubled cooling t8/5 time in the case of hybrid welding resulted in the content of the bainitic phase having a much larger share in the joint (reaching 97% in some areas), compared to a maximum of 32% in the case of laser welding. Such a distribution of metallurgical phases and cooling rates was reflected in the hardness distributions calculated on the cross-section of the analyzed joints. A comparison of the results of numerical analyses with the results of measurements on real samples again showed the high conformity of the results of calculations with the reality. 

The higher content of the martensitic structure in the laser welded joint also had an effect on the calculated distributions and von Mises stress values in the analyzed joints. Maximum values in the laser welding were higher by almost 50 MPa than in the hybrid welding case. However, similarly and characteristically, the maximum stresses were distributed in both cases in the HAZ and the adjacent zone. The distribution of residual stresses looked similar. The zone of maximum tensile stress was located in the HAZ area, and the maximum compressive stress occurred in the area under the stitch where, in the case of hybrid welding, the melting was done only as the result of the laser beam influence. Calculated values of the cumulative plastic strain distribution indicate the areas of strain changed due to the influence of thermal cycles. These results can be used by engineers as a parameter for mechanical characteristics of material changes. In general, it can be said that it is a cumulative measure of how much yielding strain occurs. In further engineering considerations, they can be used as a failure criterion at low cycle fatigue.

To sum up, using numerical simulations, engineers can very easily and quickly check what will happen when they change process parameters. This is a particularly significant fact, whereby we do not incur the costs associated with carrying out real welding tests, which, from the economic point of view, are becoming less and less profitable. It is also possible to achieve very detailed information about the process parameters and results which changed during the time of processing.

## Figures and Tables

**Table 1:** Chemical composition and typical properties of S700MC steel according to the EN10149-2 standard.

**Chemical Composition (wt.%)**															
**C**	**Mn**	**Si**		**P**	**S**		**Al**	**Nb**		V *		Ti *	**Mo**		**B**
≤0.12	≤2.1	≤0.6		≤0.025	≤0.015		≥0.015	≤0.09		≤0.2		≤0.22	≤0.5		≤0.005
**Mechanical Properties (Parallel to the Rolling Direction)**															
Minimum yield strength R_e_ (MPa)			Minimum–maximum tensile strength R_m_ (MPa)			Elongation					Temperature (°C)			Notch impact energy (J)	
						Total elongation A80% < 3 mm			Total elongation A5% ≥ 3 mm						
700			750–950			≥10			≥12		−20			≥27	

* The sum of Nb, V, and Ti being <0.22 is guaranteed according to EN10149-2.

**Table 2:** Laser T-joint welding parameters of S700MC steel plates with a thickness of 10 mm. Remarks: gas shielding, argon; gas flow rate, 20 dm^3^/min; preheating drying temperature, 65 °C.

Joint Designation	Laser Beam Power (W)	Welding Speed (m/min)	Laser Beam Focus Position (mm)	Quality Assessment
LAS1	6000	2.0	−4	High quality, no visual defects
LAS2			−6	
LAS3	7000		−4	
LAS4			−6	
LAS5			−8	
LAS6	8000		−6	
LAS7	11000		−8	

**Table 3:** Parameters for hybrid laser arc welding (HLAW) (laser beam—GMAW) T-joints. Remarks: gas shielding, argon; gas flow rate, 20 dm^3^/min; preheating drying temperature, 65 °C; arc heat source was placed 4 mm behind the laser beam.

Joint Designation	Laser Beam Power (W)	Welding Current (A)	Welding Speed (m/min)	Quality Assessment
HYB1	8500	280	2.0	High quality, small angular plate displacement
HYB2	7000	295		High quality, no visual defects
HYB3	7000	295		
HYB4	7000	290		
HYB5	7600	290		Low quality, penetration throughout with liquid metal leakage along the entire length of the joint
HYB6	8500	290		

**Table 4:** Chemical composition and mechanical properties of filler metal GMn4Ni1.5CrMo.

**Chemical Composition (wt.%)**									
C	Mn		Si	Cr		Ni	Mo		Ti
0.1	1.8		0.7	0.3		2.0	0.55		0.07
**Mechanical Properties**									
Tensile Strength Rm (MPa)		Yield Point Re (MPa)			Elongation A_5_ (%)			Impact Strength (J/cm^2^) at −40 °C	
900		810			18			55	

**Table 5:** Welding parameters used in welding simulations. cooling medium: free air in temperature 20°C, preheating temperature: 65°C.

Process	EPUL (J/mm)	v (mm/s)	k	Heat Source Model Parameters *
Laser	330	33.3	0.8	2.5/2.0/8.0
Hybrid (laser)	250		0.8	2.5/2.0/9.0
Hybrid (MAG)	230		0.8	9.0/7.0/3.0

EPUL—energy per unit length, v—welding speed, k—welding method efficiency factor; * heat source model parameters (in SYSWELD): for 3D conical model (laser)—top diameter of weld/bottom diameter of weld/penetration; for double-ellipsoidal model (MAG)—molten pool length/molten pool width/penetration.

**Table 6:** Comparison of measured and calculated hardness values in laser and hybrid welding of S700MC steel T-joints with a thickness of 10 mm. HAZ—heat-affected zone; FEM—finite element modeling.

Vickers Hardness HV1 (Test Force–9.807 N)															
Method	Base Material			HAZ			Weld			HAZ			Base Material		
**Real Welding Measurements**															
Laser	280	281	279	281	287	290	338	350	347	288	283	284	280	283	278
Hybrid	278	282	281	271	263	269	299	303	301	272	268	269	278	283	280
**Calculated Values (FEM)**															
Laser	278	274	274	253	254	268	343	343	342	254	269	258	273	274	278
Hybrid	278	275	274	268	272	276	294	293	292	276	278	268	274	274	278
